# Incidence and long-term specific mortality trends of metabolic syndrome in the United States

**DOI:** 10.3389/fendo.2022.1029736

**Published:** 2023-01-17

**Authors:** Weiya Li, Xinfan Qiu, Huan Ma, Qingshan Geng

**Affiliations:** Guangdong Cardiovascular Institute, Guangdong Provincial People’s Hospital, Guangdong Academy of Medical Sciences, Guangzhou, China

**Keywords:** metabolic syndrome, trend, NHANES, mortality, incidence

## Abstract

**Purpose:**

Metabolic syndrome (MetS) is extremely prevalent and related to severe diseases and death. This study aims to investigate the incidence and mortality trends among MetS over the past few decades. The gender and age differences of MetS are also explored.

**Patients and methods:**

Adults with MetS were screened in the National Health and Nutrition Examination Survey (NHANES) from 1999 to 2014. The mortality data were also acquired. Then we assessed the incidence and mortality trends of MetS in the United States.

**Results:**

Our study included 14171 participants with a mean age of 46.8 ± 19.3 years, of whom 7354 (51.9%) were women. Among them, 4789 participants were subsequently diagnosed with MetS. From 1999 to 2014, the overall trend of MetS incidence increased (from 27.6 to 32.3%; adjusted odds ratios [aOR], 1.71; 95% confidence interval [CI], 1.42-2.05; *P*-value <0.001, P for trend <0.001). In more detail, the incidence of MetS rose first but subsequently plateaued and declined. Obvious downward trends were observed from 29.6 to 2.7% for all-cause mortality (aOR, 0.12; 95%CI, 0.07-0.21; *P*-value <0.001, P for trend <0.001) and 4.8 to 0.8% for cardio-cerebrovascular mortality (aOR, 0.17; 95%CI, 0.05-0.61; *P*-value =0.007, P for trend <0.001). All-cause mortality decreased yearly, whereas cardio-cerebrovascular death increased briefly before declining and stabilizing. Similarly, the temporal mortality trends in MetS patients of different ages and genders had the same results. Specifically, the incidence of MetS was higher in women than in men (adjusted P =0.003; OR, 1.14; 95%CI, 1.05-1.24), but the mortality was significantly lower after an average of 7.7 years of follow-up (all-cause mortality, adjusted P <0.001; hazard ratio [HR], 0.68; 95%CI, 0.57-0.81; cardio-cerebrovascular mortality, adjusted P =0.004; HR, 0.55; 95%CI, 0.37-0.83).

**Conclusion:**

From 1999 to 2014, the incidence of MetS in U.S. adults significantly increased overall, while the mortality rate of MetS had a considerable downward trend. Both trends showed marked gender differences, being more prevalent and at lower risk in women compared with men. It is important to identify the factors that will curb the incidence of MetS and decrease mortality, especially in male patients.

## 1 Introduction

Metabolic syndrome (MetS) is a group of disorders that include abdominal obesity, insulin resistance, hypertriglyceridemia, low high-density lipoproteins (HDL), and arterial hypertension (1). Numerous studies have demonstrated that MetS is a cluster of interrelated risk factors associated with cancer, stroke, diabetes, and other comorbidities (2–4). Even in the general population, MetS is believed to be an indicator marker for the development of cardiovascular (CV) events (4, 5).

Along with modern risk factors for heart metabolism such as population aging, unhealthy lifestyles characterized by physical inactivity, and poor diet (6, 7). It has been estimated that the incidence of MetS affects 20-30% of the adult population globally (8), causing considerable health, social, and economic burdens. According to the findings of the 2010 Global Burden of Disease (GBD), high blood pressure, high total cholesterol, a high body mass index, and high fasting plasma glucose, respectively, account for 53%, 29%, 23%, and 16% of global disability-adjusted life years (9).

MetS is also associated with an increase in premature deaths (10–12). Previous studies have indicated that individuals with MetS were three times more likely to suffer a stroke or heart attack and two times more likely to die from these conditions compared with individuals without MetS (13). However, the annual trends of mortality in patients with MetS have not been reported. In addition, the duration of incidence in MetS trends in recent decades remains unclear (8, 14–16). Hence, we performed an updated investigation utilizing the NHANES data to explore long-term trends in MetS incidence and mortality among U.S. adults between 1999 and 2014.

Previous research has demonstrated significant gender and age differences in the prevalence and prognosis of MetS, while the gender and age differences in long-term trends have rarely been described. The Committee of the European Parliament recently recommended including gender differences in the policy planning, delivery, and monitoring of health services, citing gender and age as unique and important clinical demographic characteristics (17). Age and gender are also the two most important factors contributing to the increasing prevalence of MetS from a pathogenetic point of view (18). Moreover, as a result of the acceleration of population aging, the amount of elderly population is gradually rising. MetS incidence and mortality are several times higher in the elderly population than in the younger population (17, 19). Attention to gender medicine is a key requirement for the improvement of health strategies. Refining the assessment of age-stratified risk in patients with MetS will also facilitate the treatment of high-risk groups with greater precision. Therefore, gender and age differences in incidence and mortality in MetS patients have also been explored as a secondary objective. Our study may provide essential information for policymakers, clinicians, and concerned stakeholders in the U.S. so as to better manage MetS and improve prognosis.

## 2 Material and methods

### 2.1 Study design and participants

**Figure 1:**
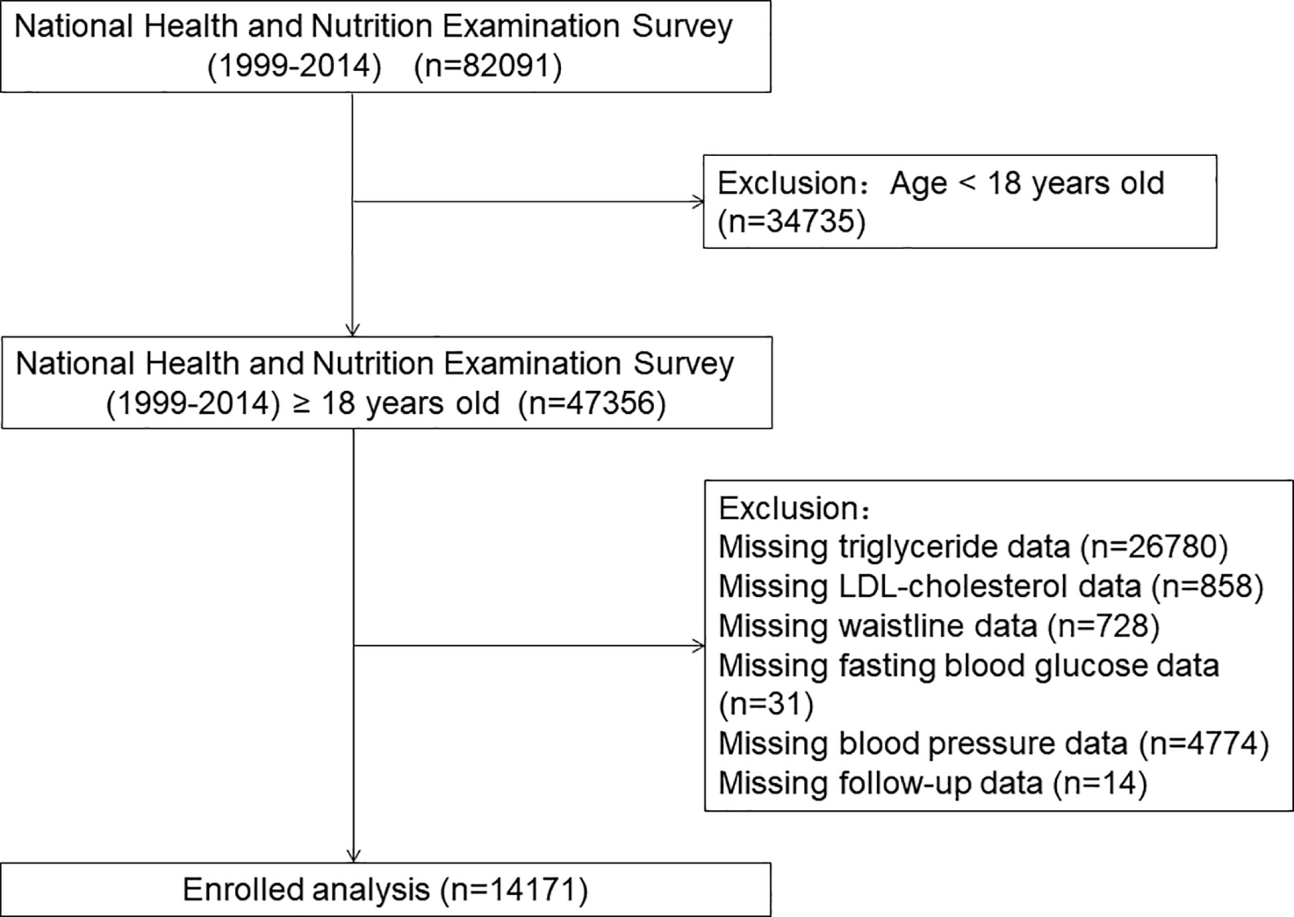
The research flow chart.

Our study aimed to investigate the incidence and mortality trends of MetS in the U.S. general adult population from 1999 to 2014. The National Health and Nutrition Examination Survey (NHANES) is a series of cross-sectional research studies conducted every two years to monitor the health of the U.S. population. The study protocols were approved by the National Center for Health Statistics (NCHS) institutional review board, and all the participants signed a written informed consent (20, 21). Well-trained medical personnel, modern testing equipment, medical reports, and the economic compensation participants received all enhance the credibility of NHANES data. Anyone can obtain details on enrollment, procedures, and population characteristics for NHANES by visiting https://www.cdc.gov/nchs/nhanes/index.htm. After excluding participants due to the lack of relevant and necessary medical data, a total of 14171 individuals, among them 4789 patients with MetS, entered the final analysis **(** **Figure 1** **)**.

### 2.2 MetS covariates and assessment

We divided educational levels into three categories (high school or less; some college; college graduate or above). “Non-Hispanic White”, “Mexican American”, “Non-Hispanic Black”, and “Other” were the categories used to describe race. A medical history of cardiovascular disease was defined as suffering from coronary heart disease, heart failure, or angina pectoris. The family poverty income ratio (PIR), calculated by dividing family income by the poverty guidelines issued by the Department of Health and Human Services (DHHS), was used to assess poverty levels. The Federal Register used each year’s DHHS poverty guidelines to determine financial eligibility for certain federal programs. We used a simple criterion and considered a family income index of less than 100% to be below the poverty line, while a ratio of 100% or higher was defined as being above the poverty line. In our study, PIR was divided into five levels (<100%; 100% -199%; 200% -299%; 300% -399%; ≥400%) (22). The criteria of the National Cholesterol Education Program (NCEP) Adult Treatment Panel III (ATP III) were commonly used worldwide, and we used this criterion to assess whether the participants had MetS (23).

MetS is diagnosed in adults when three or more of the following criteria are met: a waist circumference of ≥102 cm in men and ≥88 cm in women, high circulating triglycerides ≥150 mg/dL, low HDL <40 mg/dL for men and <50 mg/dL for women, high fasting blood glucose ≥100 mg/dL, and a diagnosis of arterial hypertension (1).

### 2.3 Follow-up and outcomes

The average follow-up time for MetS participants in this study was about 7.7 years. All-cause, cardiovascular, and cerebrovascular mortality were included as endpoints. We defined cardio-cerebrovascular mortality (I00-I09, I11, I13, I20-I51 for cardiovascular mortality; I60-I69 for cerebrovascular mortality) according to the International Classification of Diseases, 10th Clinical Modification (ICD-10) system codes. Based on a probabilistic match, the mortality data was linked from NHANES to death certificate data in the National Death Index by NCHS. Survival status has been ascertained from other sources, including links to administrative data from the Social Security Administration and the Centers for Medicare & Medicaid Services. Those with a follow-up time of 100 years or more were considered lost data and ineligible for mortality analyses. On average, 94.8% of survey participants were eligible for the mortality follow-up. The following website can be visited to learn more details about mortality variables: (https://www.cdc.gov/nchs/data-linkage/mortalitypublic.htm).

### 2.4 Statistical analysis

According to the years included, all participants were divided into eight groups (1999 through 2000, 2001 through 2002, 2003 through 2004, 2005 through 2006, 2007 through 2008, 2009 through 2010, 2011 through 2012, and 2013 through 2014). All baseline characteristics were summarized as mean ± SD, number, and percentage, or median when appropriate. Comparisons among the eight groups were made by one-way analysis of variance (ANOVA), and Pearson chi-squared tests were used for categorical variables.

Every two years from 1999 to 2014, we analyzed the incidence of MetS. The odds ratio (OR) and 95% confidence interval (CI) for MetS were calculated using single-factor and multi-factor logistic regression analyses. The all-cause and cardio-cerebrovascular mortality rates were used during an average of 7.7 years of follow-up to assess the prognostic trends in MetS patients. We used Cox regression models to investigate the temporal trend of mortality from 1999 to 2014. Models 1, 4, and 7 were not adjusted for any covariates; after adjusting for age and gender, we obtained Models 2, 5, and 8; Models 3, 6, and 9 were adjusted for multivariate variables like demographics (age, gender, ethnicity), medical and social history (education level, poverty grade, cardiovascular or cerebrovascular disease history, smoking), and laboratory examinations (CCR, hemoglobin A1c). The graphs show unadjusted and adjusted ORs (aOR), hazard ratios (HR), and 95% CI for the incidence and mortality of MetS over this time period.

We conducted additional subgroup analyses to observe the incidence and mortality trends of MetS in different age (≥65 and <65 years old) and gender (men and women) groups. In the exploratory analysis, we employed binary logistic regression to investigate the gender difference in MetS among all participants over these 16 years, using a COX proportional risk model to analyze the gender and age differences in mortality among all MetS patients. All analyses were performed with SPSS version 25, and a P value <0.05 was considered statistically significant.

## 3 Results

### 3.1 Baseline characteristics

Between 1999 and 2014, a total of 14171 participants from the NHANES were included in this study. Among them, 4789 individuals were subsequently diagnosed with MetS. The average follow-up time was 92.9 ± 51.2 months. Overall, the mean age was 46.8 ± 19.3 years old, and 7354 (51.9%) of the participants were women. In patients with MetS, the ratios regarding unqualified waist, high circulating triglycerides, low HDL, high fasting blood glucose, and diagnosis of arterial hypertension were 87.7%, 54.3%, 78.3%, 24.7%, and 80.7%, respectively. All data about baseline characteristics are described in **Table 1**.

**Table 1:** Characteristics of included general adults in NHANES from 1999–2002 to 2011–2014.

**Characteristic**	**Overall**	**1999-2000**	**2001-2002**	**2003-2004**	**2005-2006**	**2007-2008**	**2009-2010**	**2011-2012**	**2013-2014**	*P*-value
	**N=14171**	**N=1506**	**N=1659**	**N=1670**	**N=1663**	**N=1872**	**N=2077**	**N=1806**	**N=1918**	
Demographic										
Age, years	46.8 ± 19.3	45.5 ± 20.0	45.5 ± 19.8	46.3 ± 20.7	45.2 ± 20.1	48.9 ± 18.6	47.7 ± 18.7	46.9 ± 18.3	47.9 ± 18.3	<0.001
Gender, n(%)										0.158
Women	7354(51.9)	686(45.6)	798(48.1)	813(48.7)	808(48.6)	932(49.8)	978(47.1)	901(49.9)	901(47.0)	
Men	6817(48.1)	820(54.4)	861(51.9)	857(51.3)	855(51.4)	940(50.2)	1099(52.9)	905(50.1)	1017(53.0)	
Race, n(%)										<0.001
Non-Hispanic White	6469(45.6)	635(42.2)	856(51.6)	824(49.3)	815(49.0)	862(46.0)	959(46.2)	676(37.4)	842(43.9)	
Mexican American	2743(19.4)	467(31.0)	385(23.2)	365(21.9)	320(19.2)	335(17.9)	428(20.6)	185(10.2)	258(13.5)	
Non-Hispanic Black	2860(20.2)	268(17.8)	303(18.3)	363(21.7)	401(24.1)	380(20.3)	341(16.4)	429(23.8)	375(19.6)	
Other	2099(14.8)	136(9.0)	115(6.9)	118(7.1)	127(7.6)	295(15.8)	349(16.8)	516(28.6)	443(23.1)	
BMI, kg/m2	28.3 ± 6.5	27.8 ± 6.2	27.7 ± 6.1	28.0 ± 6.2	28.5 ± 6.7	28.2 ± 6.2	28.7 ± 6.7	28.5 ± 6.6	28.8 ± 7.2	<0.001
Social history										
Educational level, n(%)										<0.001
High school or less	6624(46.7)	790(59.4)	762(51.1)	798(53.9)	759(51.1)	951(53.6)	1006(51.1)	763(44.4)	795(43.9)	
Some college	3589(25.3)	313(23.6)	436(29.3)	399(27.0)	421(28.4)	450(25.4)	546(27.7)	482(28.0)	542(30.0)	
College graduate or above	2841(20.0)	226(17.0)	292(19.6)	283(19.1)	304(20.5)	373(21.0)	417(21.2)	474(27.6)	472(26.1)	
Smoking≥100 cigarettes in life, n(%)										<0.001
No	7082(50.0)	700(52.7)	756(50.7)	753(50.8)	749(50.5)	928(52.3)	1085(55.0)	991(57.7)	1120(58.4)	
Yes	6085(42.9)	629(47.3)	734(49.3)	728(49.2)	734(49.5)	848(47.7)	888(45.0)	727(42.3)	797(41.6)	
Poverty income ratio, n(%)										<0.001
<100%	2773(19.6)	254(19.4)	277(17.9)	334(21.1)	290(18.3)	355(20.6)	432(22.8)	408(24.6)	423(23.7)	
100%-199%	3403(24.0)	330(25.3)	385(24.8)	425(26.9)	399(25.1)	441(25.6)	545(28.8)	441(26.6)	437(24.5)	
200%-299%	1971(13.9)	199(15.2)	246(15.9)	253(16.0)	247(15.6)	299(17.4)	264(14.0)	215(13.0)	248(13.9)	
300%-399%	1567(11.1)	171(13.1)	190(12.3)	175(11.1)	231(14.5)	201(11.7)	201(10.6)	190(11.5)	208(11.7)	
≥400%	3368(23.8)	352(27.0)	453(29.2)	395(25.0)	421(26.5)	425(24.7)	449(23.7)	405(24.4)	468(26.2)	
Medical history										
Cardiovascular disease	1018(7.2)	81(6.1)	111(7.5)	132(9.0)	113(7.7)	161(9.2)	153(7.8)	126(7.4)	141(7.8)	0.072
Stroke, n (%)	447(3.2)	29(2.2)	42(2.8)	55(3.7)	60(4.0)	67(3.8)	65(3.3)	68(4.0)	61(3.4)	0.093
cardio-cerebrovascular disease, n (%)	1291(9.1)	99(7.5)	133(9.0)	167(11.4)	147(10.0)	204(11.6)	193(9.9)	168(9.8)	180(10.0)	0.008
Laboratory examination										
CCR, mg/min	118.8 ± 51.0	147.7 ± 68.0	115.2 ± 50.0	114.2 ± 47.3	115.5 ± 48.0	114.5 ± 45.4	116.8 ± 47.2	116.9 ± 46.5	114.6 ± 48.3	<0.001
HbA1c,%	5.6 ± 1.0	5.4 ± 0.9	5.5 ± 0.9	5.5 ± 0.9	5.5 ± 1.0	5.7 ± 1.1	5.7 ± 0.9	5.7 ± 1.1	5.7 ± 1.0	<0.001

BMI, Body Mass Index; CCR, Creatinine Clearance Rate; HbA1c, hemoglobin A1c.

In addition, the incidence of MetS had a reverse association with the level of education (r =- 0.076, p <0.001). MetS was positively correlated with a history of cardiovascular disease (r =0.062, p <0.001) and smoking ≥100 cigarettes (r =0.101, p <0.001).

### 3.2 Trends in the incidence of MetS

**Figure 2:**
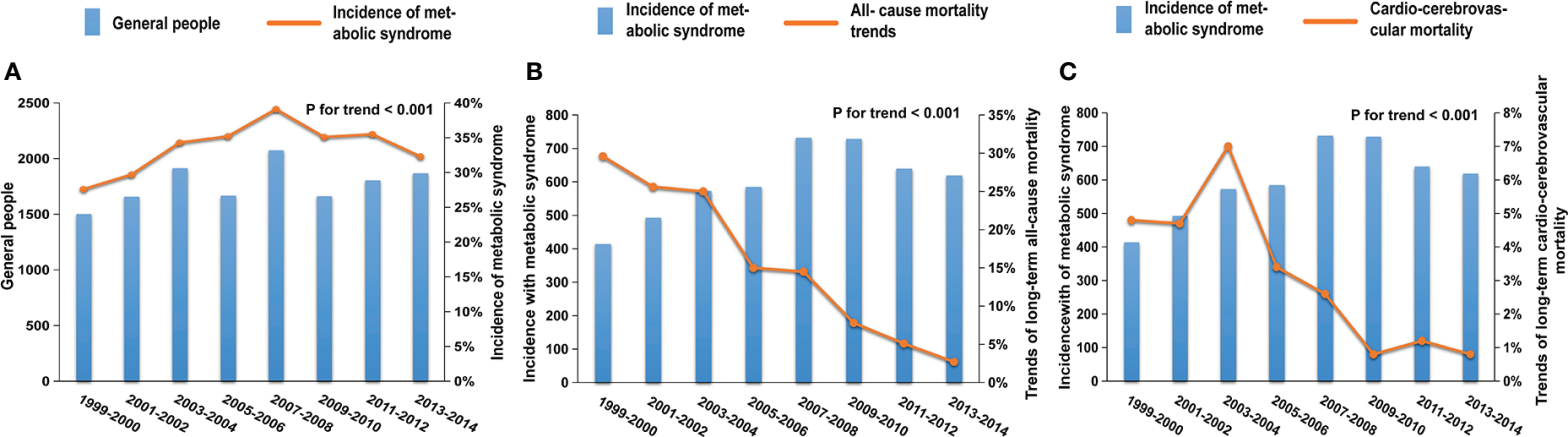
Trends in the incidence and mortality of MetS patients in the NHANES from 1999 to 2014; **(A)** The prevalence of MetS increased significantly from 27.6 to 32.3% (adjusted odds ratios [aOR], 1.71; 95%CI, 1.42-2.05; P-value <0.001, P for trend <0.001); trends in all-cause **(B)** and cardio-cerebrovascular **(C)** mortality of MetS patients in the NHANES from 1999 to 2014. After an average of 7.7 years of follow-up, the all-cause mortality (from 29.6 to 2.7%; [aOR], 0.12; 95%CI, 0.07-0.21; P-value <0.001, P for trend <0.001) and cardio-cerebrovascular mortality (from 4.8 to 0.8%; [aOR], 0.17; 95%CI, 0.05- 0.61; P-value =0.007, P for trend <0.001) of MetS patients showed obvious downward trends.

From 1999-2000 to 2013-2014, a total of 4789 (33.8%) participants in the general population developed MetS. Compared with 1999-2000, MetS incidence in 2013-2014 increased significantly, from 27.6 to 32.3% (adjusted odds ratios [aOR], 1.71; 95% CI, 1.42–2.05; *P*-value <0.001, P for trend <0.001) (**Figure 2A**; **Table 2**). The incidence of MetS displayed a significant upward trend over the course of 16 years. In all MetS patients, unqualified waist, low HDL, and high fasting blood glucose contributed the most to MetS, with a total percentage of 87.7%, 78.3%, and 80.7% respectively. As for each component of the MetS separately, the unqualified waist had a slight and small upward trend from 87.5 to 91.1% overall. Both low HDL and high fasting blood glucose had a significant upward trend from 70.4 to 77.7% and 70.4 to 85.6%, respectively, while high circulating triglycerides decreased from 66 to 44.1%, and arterial hypertension went from 30.1 to 24.2%.

**Table 2:** Odds ratios for prevalence and hazard ratios for all-cause, cardiovascular, and cerebrovascular mortality from 1999-2002 to 2011-2014.

Odds ratios for the prevalence of MetS				MetS all-cause mortality hazard ratios				MetS cardiovascular and cerebrovascular mortality hazard ratios			
Years	Odds ratios	*P*-value	P for trend	Years	Hazard ratios	*P*-value	P for trend	Years	Hazard ratios	*P*-value	P for trend
Model 1				Model 4				Model 7			
1999-2000	1(reference)	–	<0.001	1999-2000	1(reference)	–	<0.001	1999-2000	1(reference)	–	<0.001
2001-2002	1.11(0.95-1.30)	0.18		2001-2002	1.00(0.78-1.28)	0.969		2001-2002	1.07(0.59-1.95)	0.823	
2003-2004	1.37(1.18-1.60)	<0.001		2003-2004	1.03(0.81-1.32)	0.811		2003-2004	1.67(0.97-2.87)	0.066	
2005-2006	1.43(1.23-1.66)	<0.001		2005-2006	0.85(0.64-1.12)	0.238		2005-2006	1.13(0.60-2.12)	0.703	
2007-2008	1.69(1.46-1.95)	<0.001		2007-2008	0.08(0.06-0.11)	<0.001		2007-2008	0.09(0.04-0.17)	<0.001	
2009-2010	1.43(1.23-1.65)	<0.001		2009-2010	0.06(0.04-0.08)	<0.001		2009-2010	0.04(0.01-0.09)	<0.001	
2011-2012	1.45(1.25-1.68)	<0.001		2011-2012	0.08(0.05-0.11)	<0.001		2011-2012	0.10(0.04-0.23)	<0.001	
2013-2014	1.25(1.08-1.45)	0.003		2013-2014	0.11(0.07-0.18)	<0.001		2013-2014	0.20(0.07-0.53)	0.001	
Model 2	1(reference)			Model 5				Model 8			
1999-2000		–	<0.001	1999-2000	1(reference)	–	<0.001	1999-2000	1(reference)	–	<0.001
2001-2002	1.13(0.95-1.30)	0.145		2001-2002	1.15(0.89-1.47)	0.282		2001-2002	1.31(0.72-2.40)	0.382	
2003-2004	1.38(1.18-1.62)	<0.001		2003-2004	0.97(0.76-1.24)	0.821		2003-2004	1.60(0.93-2.74)	0.09	
2005-2006	1.51(1.28-1.76)	<0.001		2005-2006	0.79(0.60-1.04)	0.098		2005-2006	1.07(0.57-2.00)	0.834	
2007-2008	1.60(1.37-1.90)	<0.001		2007-2008	0.14(0.11-0.18)	<0.001		2007-2008	0.18(0.09-0.34)	<0.001	
2009-2010	1.38(1.19-1.60)	<0.001		2009-2010	0.10(0.07-0.14)	<0.001		2009-2010	0.07(0.03-0.18)	<0.001	
2011-2012	1.45(1.24-1.69)	<0.001		2011-2012	0.12(0.08-0.24)	<0.001		2011-2012	0.19(0.08-0.45)	<0.001	
2013-2014	1.20(1.02-1.40)	0.023		2013-2014	0.14(0.09-0.24)	<0.001		2013-2014	0.29(0.11-0.80)	0.016	
Model 3				Model 6				Model 9			
1999-2000	1(reference)	–	<0.001	1999-2000	1(reference)	–	<0.001	1999-2000	1(reference)	–	<0.001
2001-2002	1.71(1.41-2.07)	<0.001		2001-2002	1.14(0.85-1.52)	0.66		2001-2002	1.37(0.70-2.70)	0.359	
2003-2004	2.20(1.82-2.65)	<0.001		2003-2004	0.81(0.61-1.06)	0.103		2003-2004	1.32(0.71-2.46)	0.386	
2005-2006	2.47(2.04-2.98)	<0.001		2005-2006	0.67(0.49-0.91)	0.008		2005-2006	0.80(0.39-1.65)	0.548	
2007-2008	2.33(1.94-2.79)	<0.001		2007-2008	0.13(0.10-0.18)	<0.001		2007-2008	0.15(0.07-0.32)	<0.001	
2009-2010	1.88(1.57-2.25)	<0.001		2009-2010	0.10(0.07-0.14)	<0.001		2009-2010	0.06(0.02-0.18)	<0.001	
2011-2012	2.15(1.78-2.59)	<0.001		2011-2012	0.09(0.06-0.14)	<0.001		2011-2012	0.13(0.04-0.36)	<0.001	
2013-2014	1.71(1.42-2.05)	<0.001		2013-2014	0.12(0.07-0.21)	<0.001		2013-2014	0.17(0.05-0.61)	0.007	

Model 1, Model 4, and Model 7: unadjusted.

Model 2, Model 5, and Model 8: adjusted for age and gender.

Model 3, Model 6, and Model 9: adjusted for multivariate variables (age, gender, education level, ethnicity, cardiovascular and cerebrovascular disease history, CCR, poverty grade, HbA1c, smoking≥100 cigarettes).

### 3.3 Trends of all-cause and cardio-cerebrovascular mortality in MetS patients

During the average of 7.7 years of follow-up, there were a total of 693 (14.5%) and 141 (2.9%) patients with MetS who experienced all-cause and cardio-cerebrovascular death, respectively. Compared with 1999-2000, the all-cause mortality rate in 2013-2014 significantly decreased from 29.6 to 2.7% (**Figure 2B**; **Table 2**; adjusted odds ratios [aOR], 0.12; 95%CI, 0.07-0.21; *P*-value <0.001, P for trend <0.001), and the cardio-cerebrovascular mortality rate in 2013-2014 significantly decreased from 4.8 to 0.8% (**Figure 2C**; **Table 2**; adjusted odds ratios [aOR], 0.17; 95% CI, 0.05-0.61; *P*-value =0.007, P for trend <0.001).

### 3.4 Subgroup analysis

**Figure 3:**
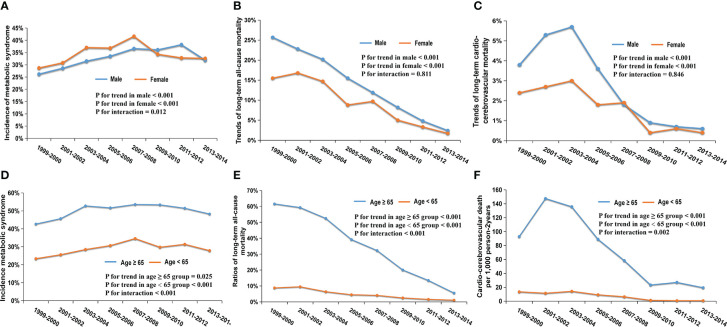
Trends in the incidence and mortality of different gender and age subgroups from 1999 to 2014; **(A)** Trends in the incidence of MetS among men (P for trend <0.001) and women (P for trend <0.001); **(B)** Trends of 7.7-year all-cause mortality among men (P for trend<0.001) and women (P for trend <0.001); **(C)** Trends of 7.7-year cardio-cerebrovascular mortality among men (P for trend<0.001) and women (P for trend <0.001); **(D)** Trends in the incidence of MetS among ≥65 years (P for trend=0.025) and <65 years (P for trend <0.001); **(E)** Trends of 7.7-year all-cause mortality among ≥65 years (P for trend<0.001) and <65 years (P for trend <0.001); **(F)** Trends of 7.7-year cardio-cerebrovascular mortality among ≥65 years (P for trend<0.001) and <65 years (P for trend <0.001).

In our study, 2260 (47.2%) men and 2529 (52.8%) women were diagnosed with MetS. The incidence of MetS displayed an upward trend in both groups (**Figure 3A**; men: 26.2 to 31.9%, P for trend <0.001; women: 28.7 to 32.6%, P for trend <0.001, P for interaction = 0.012).

There were 371 (16.4%) all-cause deaths in men and 322 (12.7%) in women during the period of follow-up among patients with MetS. In general, a downward trend in all-cause mortality appeared in two gender groups (**Figure 3B**; men: 25.7 to 2.4%, P for trend <0.001; women: 15.5 to 1.7%, P for trend <0.001; P for interaction = 0.598). 80 men (3.5%) and 61 women (2.4%) experienced cardio-cerebrovascular death in patients with MetS. The cardio-cerebrovascular mortality of the two groups both displayed a significant downward trend over 16 years (**Figure 3C**; men: 3.8 to 0.6%, P for trend <0.001; women: 2.4 to 0.4%, P for trend <0.001; P for interaction = 0. 811).

From 1999-2000 to 2013-2014, 1570 (50.3%) participants older than 65 developed MetS, which was significantly higher than 3219 (29.1%) of patients <65. The risk of suffering from MetS in the two groups showed an upward trend (**Figure 3D**; ≥ 65-year-old group, 42.7 to 48.3%, P for trend = 0.025; <65-year-old group, 23.4 to 27.9%, P for trend < 0.001; P for interaction = 0.846).

All-cause death occurred in 493 (31.4%) MetS patients in the ≥65-year-old group and 200 (6.2%) in the <65- year-old-group, respectively. Both groups showed a downward trend in all-cause mortality (**Figure 3E**; ≥65 group: 61.6 to 5.6%, P for trend <0.001; <65 group: 8.8 to 1.1%, P for trend <0.001; P for interaction <0.001). Cardio-cerebrovascular death occurred in 107 (6.8%) patients over the age of 65, while it occurred in 34 (1.1%) patients <65. Due to insufficient death samples, we used per 1000 person-2 years to describe mortality. There were downward trends in both groups, (**Figure 3F**; ≥65 group, 92.9 to 19.4 per 1000 person-2 years, P for trend < 0.001; <65 group, 13.5 to 0.7 per 1000 person-2 years, P for trend <0.001; P for interaction =0.002). Interestingly, the downward trends in mortality in these two age groups had statistically significant differences (both P for interaction < 0.05).

### 3.5 Exploratory analysis

We conducted further analysis to explore the differences in incidence and mortality between different genders and ages over 16 years. Overall, there were 2529 (52.8%) women and 2260 (47.2%) men who developed MetS. There were 3219 (29.1%) participants aged <65 years and 1570 (50.3%) participants aged≥65 years who were diagnosed with MetS. In the binary logistic regression, women had a higher incidence than men after adjustment of multivariate variables (age, education level, ethnicity, cardiovascular or cerebrovascular disease history, CCR, poverty grade, HbA1c, smoking ≥100 cigarettes) (adjusted P= 0.003; OR, 1.14; 95% CI, 1.05-1.24). Older participants (≥65 years) had a higher incidence of MetS than that in the younger group (≥65 years) after adjustment of multivariate variables (gender, education level, ethnicity, cardiovascular or cerebrovascular disease history, CCR, poverty grade, HbA1c, smoking ≥100 cigarettes) (adjusted P <0.001; OR, 2.58; 95%CI, 2.30-2.89).

**Figure 4:**
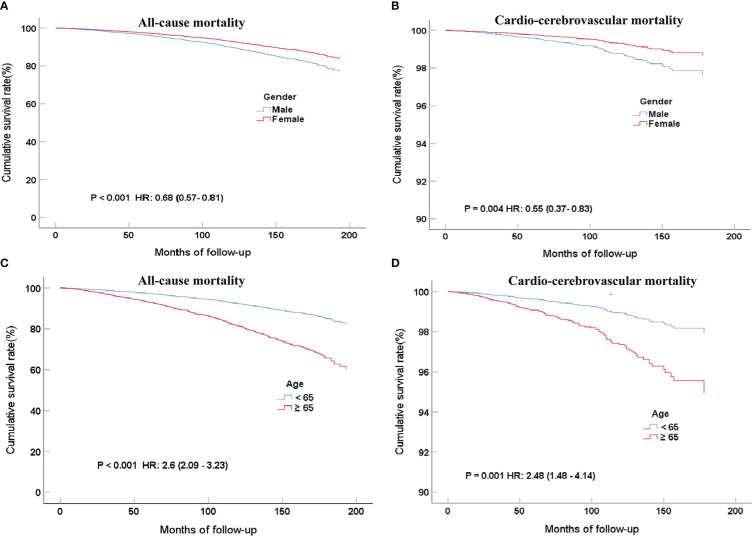
COX regression curves for all-cause **(A)** and cardio-cerebrovascular **(B)** mortality rates of MetS patients grouped by gender after multi-factor adjustment and 7.7 years of follow-up; COX regression curves for all-cause **(C)** and cardio-cerebrovascular **(D)** mortality rates of MetS in different age groups after multi-factor adjustment and 7.7 years of follow-up.

Unexpectedly, women had a lower risk than men, no matter if it was all-cause (16.4% vs. 12.7%) or cardio-cerebrovascular mortality (3.5% vs. 2.4%) after a long-term follow-up. In the COX proportional risk model, the mortality of men was lower than that of women and achieved statistical significance (for all-cause mortality, P <0.001; HR, 0.71; 95%CI, 0.61-0.82; For cardio-cerebrovascular mortality, P =0.006; HR, 0.76; 95%CI, 0.45-0.88). The same conclusion still existed after adjusting for multivariate variables (age, gender, education level, ethnicity, cardiovascular or cerebrovascular disease history, CCR, poverty grade, HbA1c, smoking ≥100 cigarettes) (**Figure 4A** for all-cause mortality, adjusted P <0.001; HR, 0.68; 95%CI, 0.57-0.81; **Figure 4B** for cardio-cerebrovascular mortality, adjusted P =0.004; HR, 0.55; 95%CI, 0.37-0.83). In terms of the mortality of patients with MetS, the older age group still had a higher mortality risk than the lower age group after multivariate variables were adjusted (**Figure 4C** for all-cause mortality, adjusted P <0.001; HR, 2.6; 95%CI, 2.09-3.23; **Figure 4D** for cardio-cerebrovascular mortality, adjusted P <0.001; HR, 2.48; 95%CI, 1.48-4.14).

To further explore the impact of each component of the MetS on mortality, all five factors were put into a Cox proportional regression model. And we found that high fasting blood glucose had the highest HR (2.28 for all-cause mortality and 2.79 for cardio-cerebrovascular mortality) and was the only significant factor to increase mortality (P <0.001 for all-cause mortality and P =0.001 for cardio-cerebrovascular mortality).

## 4 Discussion

To the best of our knowledge, this is the first study to explore the trends in long-term mortality over the MetS span of 16 years in the United States. Our research suggests that nearly 34% of all adults and 50% of those aged ≥65 years were estimated to have MetS and showed an upward trend from 1999 to 2014 overall. The all-cause and cardio-cerebrovascular mortality of patients with MetS were 14.5% and 2.9%, respectively. Fortunately, mortality rates showed a significant downward trend from 1999 to 2014 overall.

According to our graphics and data analysis results, with the boundary of 2008, the incidence trend of adult MetS rose before 2008 and then decreased. The same trend existed in women, regardless of the age subgroup **(** **Figures 3A, D** **)**, while in the men’s subgroup an obvious decline was observed until 2012, which had a statistically significant difference in overall trends compared with women (P for interaction =0.012; **Figure 3A**). Similar to previous studies of older adults (19, 24), half of the elderly over 65 years old suffer from MetS. This is a worrying finding since the U.S. will soon experience a massive increase in its older population (25), which may cause the prevalence of MetS to increase even more than it already has. We cannot reduce MetS vigilance due to the disease’s stable incidence in recent years, especially among the elderly.

The all-cause mortality of MetS patients showed a continuous decline, whether in general or across all subgroups (**Figures 2B**, **3B, E**). Compared with the younger MetS subgroup (<65), the older group (≥65) had a more remarkable drop that reached statistical significance (P for interaction <0.001; **Figure 3B**). The trend of cardio-cerebrovascular mortality in MetS patients continued decreasing, in addition to a brief increase in 2001-2004 (**Figures 2C**, **Figures 3C, F**). There was no statistical difference in the mortality trends between men and women. In the younger subgroup, the decreasing trend of mortality was relatively stable, while an obviously declining trend was observed in the older subgroup (P for interaction = 0.002; **Figure 3F**). More attention should be paid to the prognosis of older patients with MetS. Abdominal obesity, as an important component of MetS, still has a rising trend in the U.S (25). More emphasis on abdominal obesity may reduce mortality even further.

Interestingly, for all participants, women have a higher incidence of MetS than men, but men have a higher death ratio than women, which reached statistical significance. Future research needs to explore specific reasons for the high mortality rate of men with MetS, so as to improve their prognosis by correcting hazardous factors.

Although the incidence and mortality of MetS in different age groups showed a downward trend, the incidence and mortality of MetS in older people were significantly higher. With the aging population in the U.S., more attention should be given to medical care issues to reduce the national medical burden.

MetS is thought to be a chronic, low-grade inflammatory state caused by the complex interaction of environmental and genetic factors (13). MetS was accounted for by heritability estimates ranging between approximately 10 and 30% (26). Environmental factors such as physical inactivity, an unhealthy diet, stress, and tobacco use are also closely related to the incidence of MetS. People with low education levels appeared to be more likely to suffer from MetS, which is consistent with previous research. People with a high level of education may be more mindful of inactivity, unhealthy eating patterns, and risky behaviors. In addition, they are more likely to take care of themselves by exercising, ordering regular health check-ups, and avoiding risky behaviors such as smoking and drinking too much (27, 28). All of these reasons might explain this (29). Previous studies have shown that smoking, even at low levels (mean <30 cigarettes weekly), is associated with MetS (30–32). This conclusion is consistent with our findings. A long history of cardiovascular disease (like coronary heart disease, heart failure, and angina pectoris) was positively linked to MetS. The reason might be that cardiovascular disease is closely associated with glycolipid metabolism, hypertension, and obesity, which together make for an easier MetS diagnosis (33–37). Knowledge of the factors influencing the increasing incidence of MetS in different populations is needed to assist in the prevention of cardiovascular disease and type 2 diabetes.

Our results suggest that MetS generally exists in the general population, especially in women and the elderly. It is critical to improving prevention treatments for MetS patients with high mortality risk, particularly among the elderly and male populations. In addition to this, the incidence of MetS in women is high, but the mortality is relatively low. A higher prevalence of MetS may be attributable to physical and psychological factors. The average age of women in our study was 54.6 years old, which may mean increased abdominal obesity and a reduction in HDL-cholesterol after menopause and make it easier to meet the MetS diagnostic criteria (17). Women are also more likely than men to develop MetS because of work stress and low socioeconomic status (17). It is a long-standing belief and undeniable evidence that women are more protected from CV events than men (17). Previous research demonstrated that men with MetS had a higher risk of severe CVs and mortality than women (38, 39). Some studies supported the opposite conclusion or described men and women as having equal risk (17). The association between MetS and poor prognosis may be affected by the study design (e.g., MetS definition, duration of follow-up, level of follow-up loss, and adjustment for covariates), study subjects (e.g., race, gender, and pre-morbid conditions) (40). HDL cholesterol levels and smoking were thought to play a significant role in explaining the gender difference in coronary heart disease incidence and mortality (41). In our study, men had a higher smoking rate and a higher percentage of unqualified HDL cholesterol. Men had a significantly higher prevalence of cardiovascular and cerebrovascular disease histories (16.7% vs. 12.1%, Chi-squared Test, P<0.001), indicating a worse pre-morbid condition than women. However, the exact underlying reasons merit verification through a further, rigorous prospective study.

Several limitations in our study should still be acknowledged. First, we used more stringent diagnostic criteria for MetS, which may have allowed us to underestimate its incidence while applying only objective data will reduce our MetS classification error. Second, the sample of the U.S. general population may limit its applicability to other regions and populations. Thirdly, we have not included the factors that may affect the incidence and mortality trends in MetS patients, such as exercise and nutrition. Despite all of the above, our study is important because we detailed the latest trends in MetS mortality in the United States by using a nationally representative sample. Finally, the NDI only includes deaths that occurred in the U.S. or a U.S. territory, so it may not include the deaths of all survey participants, resulting in an underestimation of mortality.

## 5 Conclusion

From 1999 to 2014, the incidence of MetS in U.S. adults considerably increased overall, while the mortality rate had a significant downward trend. Both trends showed marked gender differences, with women exhibiting greater prevalence and lower risk than men. It is important to identify the factors that will reduce MetS incidence and mortality, particularly in male patients.

## Data availability statement

The datasets presented in this study can be found in online repositories. The names of the repository/repositories and accession number(s) can be found on: https://www.cdc.gov/nchs/data-linkage/mortalitypublic.htm.

## Ethics statement

The studies involving human participants were reviewed and approved by Ethics Committee of the People’s Hospital of Guangdong Province. The patients/participants provided their written informed consent to participate in this study.

## Author contributions

WL, and XQ: conceptualization and methodology. WL and HM: formal analysis. HM and QG: supervision and validation. WL, XQ, and HM: writing and revision. All authors contributed to the article and approved the submitted version.
